# Targeting ATM to mitigate intervertebral disc degeneration

**DOI:** 10.18632/aging.203015

**Published:** 2021-04-22

**Authors:** Louise E. Pitcher, Alec K. Wong, Paul D. Robbins

**Affiliations:** 1 Institute on the Biology of Aging and Metabolism, University of Minnesota Medical School, Minneapolis, MN 55455, USA

**Keywords:** aging, DNA damage, signal transduction, senescence

Persistent DNA damage and the ensuing dysregulated cellular damage responses are implicated as the major drivers of aging and age-related diseases including intervertebral disc degeneration (IDD). Accelerated age-associated IDD occurs in the *Ercc1*^-/∆^ progeria mouse model with increased DNA damage. Ataxia telangiectasia mutated (ATM) is a protein kinase activated by DNA damage that contributes to driving cellular senescence. Heterozygosity in ATM in *Ercc1*^-/Δ^ mice was shown to reduce cellular senescence, improve stem cell function and extend healthspan. The manuscript by Han et al. extended the analysis of whether persistent DNA damage contributes to chronic dysregulated activation of ATM signaling to the intervertebral disc, increasing cellular senescence and matrix homeostatic imbalance. They demonstrate that heterozygosity in ATM leads to reduction in markers of cellular senescence, matrix aggrecanolysis, and proteoglycan loss in the intervertebral discs of *Ercc1^-/∆^* mice as well as reduced vertebral osteoporosis. Treatment of human disc nucleus pulposus cells in culture with the DNA damaging agent cisplatin also induced senescence, aggrecanolysis, and decreased total proteoglycan production, and other degenerative changes that were all reduced by treatment with an ATM inhibitor. These findings suggest that ATM is a key regulator of DNA damage-mediated disc cellular senescence and matrix perturbation and thus represents a potential therapeutic target for spine degeneration and other age-related diseases.

Age-associated intervertebral disc degeneration (IDD) is characterized by increased senescent disc cells burden, loss of proteoglycans, and perturbation of overall matrix homeostasis [1]. The maintenance of genomic integrity plays a critical role in the regulation of cellular homeostasis and overall organismal health [2]. Prolonged exposure to endogenous and exogenous genotoxic sources results in persistent and accumulated DNA damage, inducing multiple cell fates, one of which is cellular senescence. Senescent cells are metabolically active, non-dividing cells that negatively modify their local environment through a senescence associated secretory phenotype (SASP) [3]. DNA damage also is an important driver of cellular senescence in IDD, so investigating the regulation of DNA damage response (DDR) aids the elucidation of potential underlying IDD mechanisms [1].

Progeroid *Ercc1^-/∆^* mice are deficient in the ERCC1-XPF DNA repair endonuclease complex, causing these mice to accumulate DNA damage much like natural aging, but at a faster rate. The accelerated DNA damage and senescence profile in *Ercc1*^-/∆^ mice mirrors that of natural aging, making the mouse model appropriate for studying DNA damage, senescence, and aging [4]. The protein kinase ATM recruits multiprotein complexes as a primary response to genomic damage, and its hyperactivation results in the chronic activation of NF-κB signaling. NF-κB contributing to driving senescence and expression of inflammatory SASP factors. This leads to increased expression of ADAMTS5 and matrix metalloproteinases (MMPs) that cleave bonds in aggrecan core proteins, resulting in the breakdown of cartilage [5–7]. Indeed, in human nucleus pulposus cells as well as *Ercc1^-/∆^* progeroid mouse models, persistent genotoxic stress resulted in the rapid phosphorylation of ATM and concomitant increased activation of NF-κB, aggrecanolysis, decreased proteoglycan production, and increased expression of senescence markers [6]. These data indicate that the chronic activation of DDR due to genotoxic stress induces IDD pathology.

DDR-induced ATM activation promotes senescence and reduced health span, in part, by upregulating the transcriptional activity of NF-κB. The treatment of senescent *Ercc1^-/-^* mouse embryonic fibroblasts (MEFs) with ATM inhibitor KU-55933 resulted in the significant reduction of both senescent markers and SASP factors as well as the suppression of ATM mediated NF-κB activation. Furthermore, it was demonstrated that the genetic reduction of ATM in the *Ercc1^-/∆^* mice reduces cellular senescence, improves stem cell function, extends health span, and reduces certain age-related pathologies in the musculoskeletal system, demonstrating the feasibility of targeting ATM [8]. While NF-κB activity is not solely dependent on ATM activation, it is known that genotoxic stress induces ATM mediated NF-κB activation. The inducible IκB kinase (IKK) contains the regulatory subunit NF-κB essential modifier (NEMO), which is phosphorylated by ATM, post-translationally modified, and exported to the cytoplasm in an ATM-NEMO complex [8]. This eventually leads to the activation of IKKß, a catalytic subunit of IKK that activates NF-κB through the phosphorylation and subsequent degradation of its inhibitor IκB [8]. In many animal models of aging and age-associated disease where DNA damage is induced by genotoxic stress, inhibition of ATM suppresses NEMO-dependent NF-κB activation by IKK and reduces disease associated pathology. However, it is important to note that complete silencing of ATM may have detrimental effects as ATM signaling controls many downstream pathways and is required for the maintenance of chromatin integrity. Moreover, it is unknown how factors including age, gender, and health status may affect the ATM pathway. Thus, while targeting ATM as a therapeutic strategy to extend healthspan and to treat certain age-related diseases is promising, especially those diseases driven by the accumulation of DNA damage resulting in chronic hyperactivation of ATM, further research is required.
